# The associations between personality functioning (Criterion A) and pathological personality traits (Criterion B) in the alternative model for personality disorders in DSM-5: a meta-analysis

**DOI:** 10.3389/fpsyt.2025.1673139

**Published:** 2025-09-29

**Authors:** Jens C. Thimm

**Affiliations:** Department of Psychology, UiT The Arctic University of Norway, Tromsø, Norway

**Keywords:** DSM-5 AMPD, personality dysfunction, personality traits, LPFS, PID-5, FFM

## Abstract

**Introduction:**

The Alternative Model for Personality Disorders in the fifth edition of the Diagnostic and Statistical Manual of Mental Disorders (DSM-5 AMPD) requires the assessment of personality functioning (Criterion A), using the Level of Personality Functioning Scale (LPFS), and the presence of pathological personality traits (Criterion B), operationalized with the Personality Inventory for DSM-5 (PID-5). Several studies have investigated the associations between the LPFS and the PID-5 personality traits as well as the normal-range personality traits of the Five-Factor Model (FFM) of personality. The goal of the present study was to meta-analytically integrate the findings of these studies to examine the extent to which the LPFS is related to the PID-5 and FFM traits.

**Methods:**

A systematic literature search was conducted in the databases PsycINFO, Medline, Embase, and Web of Science for studies providing information about the correlations of the measures of the LPFS with versions of the PID-5 and/or measures of the FFM in adult samples. The bivariate correlations of scales measuring the LPFS with the measures of the PID-5 and FFM traits were meta-analytically pooled.

**Results:**

Data from 44 studies and 47 independent samples were identified and used in the analyses. The results showed medium-to-large weighted average correlations between the LPFS total score and the PID-5 traits, ranging from.44 (antagonism) to.64 (detachment). Overall, lower correlations were found between the LPFS and the FFM traits.

**Discussion:**

Tentative explanations for these associations are discussed, and suggestions to reduce them—including potential modifications to one or both criteria—are presented.

**Systematic review registration:**

https://osf.io/49rs7, identifier doi.org/10.17605/OSF.IO/49RS7.

## Introduction

The Alternative Model for Personality Disorders (PDs) in section III of the fifth edition of the Diagnostic and Statistical Manual of Mental Disorders (DSM-5 AMPD; 1) represents a novel approach to the diagnosis of PDs and a departure from the categorical approach based on polythetic criteria in section II of the DSM-5. The main elements of the DSM-5 AMPD are dimensional assessments of the presence and degree of impaired personality functioning (Criterion A) and pathological personality traits (Criterion B).

Criterion A requires the evaluation of the person’s personality functioning in terms of self and interpersonal functioning. In the DSM-5 AMPD, impaired personality functioning is considered the defining core of personality pathology and common across all PD types (1). The degree of impairment in personality functioning is used to determine the severity of personality pathology. It is strongly associated with different problems in life, relevant for treatment planning, and accounts for changes in PD symptomatology over time and was therefore deemed important to be included in the DSM-5 AMPD (2). The definition of personality functioning in the DSM-5 AMPD is grounded in the psychodynamic tradition (3, 4), theorizing that personality pathology originates from maladaptive representations of the self and others (5, 6). Criterion A in the DSM-5 AMPD is operationalized by the Level of Personality Functioning Scale (LPFS). The LPFS is the result of a review of instruments for the assessment of personality functioning that were available at the time of its development (5) and subsequent statistical analysis (7). In its current version, the LPFS consists of four elements (1) or domains (4, 8) of self and interpersonal functioning: identity, self-direction, empathy, and intimacy (1). According to the LPFS, identity includes the themes of self–other differentiation, the valence and stability of self-esteem, the accuracy of self-appraisal, and the capacity to experience and regulate a broad range of emotions. Self-direction refers to the pursuit of meaningful goals, the appropriateness of internal standards of behavior, and the ability for self-reflection. Empathy involves mentalizing others’ mental states, tolerating different perspectives, and understanding one’s impact on others. Finally, intimacy concerns the capacity to establish and maintain close relationships with others and mutuality in interpersonal relations (1, 8). These four elements are assumed to cover the most central features of personality pathology (5). Because they affect each other and are closely linked (1), they are not coded separately in the DSM-5 AMPD but combined into a single assessment of the level of personality functioning. Several interview-based and self-report instruments have been developed to aid the assessment of Criterion A in the DSM-5 AMPD consistent with the LPFS (9, 10).

Criterion B in the DSM-5 AMPD involves the assessment of pathological personality traits, defined as relatively stable ways of feeling, perceiving, thinking, and behaving across time and situations (1). Whereas Criterion A is concerned with the severity of personality dysfunction, the purpose of Criterion B is to specify the style in which the dysfunction is expressed and manifests itself (11). The DSM-5 AMPD trait model consists of five broad trait domains and 25 specific trait facets within the five trait domains (1). The model was developed independently from Criterion A and together with a self-report assessment instrument for its measurement. The goals were to align the model with the five PD trait domains identified by Widiger and Simonsen (12) and Harkness et al. (13) and to ensure that the traits of the DSM-IV-TR categorical PD model (14) are covered by trait facets (15, 16). The final DSM-5 AMPD personality trait model and the 220-item Personality Inventory for DSM-5 (PID-5) were the results of an iterative process in which psychometric methods (exploratory factor analysis and item response theory) were applied to refine the model and the PID-5 (15). The five trait domains of the model include negative affectivity (frequent and intense negative emotions and their behavioral and interpersonal manifestations), detachment (social or emotional detachment), antagonism (manipulative, grandiose, or hostile behavior), disinhibition (impulsive behaviors), and psychoticism (unusual or eccentric behavior and perceptions) and are described as maladaptive variants of the personality trait dimensions of the “Big Five” or Five-Factor Model (FFM) of personality (1). Psychometric studies of the PID-5 have shown adequate reliability, a replicable factor structure across different populations, and convergent validity with other personality measures (17). Abbreviated versions of the PID-5 include the 100-item PID-5-SF (18), the 36-item PID5BF+M (19), the 34-item PID5BF+ (20), and the 25-item PID-5-BF (1).

The distinction between the severity of personality pathology operationalized by the LPFS (Criterion A) and style in terms of pathology personality traits (Criterion B) in the DSM-5 AMPD has raised the question of overlap or redundancy of the two criteria (11). An early empirical study on the DSM-5 AMPD showed substantial correlations between LPFS ratings and PID-5 scores as high as .69 between LPFS identity and PID-5 negative affectivity (21). Several subsequent studies have further reported on the relationships between the measures of personality functioning consistent with the LPFS and the pathological personality traits of the DSM-5 AMPD. For example, Nysæter et al. (22) found correlations between LPFS ratings derived from a structured interview and the PID-5 trait domains ranging from .31 (antagonism) to .75 (detachment). Sleep et al. (23) reported significant correlations between all LPFS elements and the PID-5 trait domains and trait facets. Based on their own study results, Hopwood et al. (24) suggested that LPFS identity is particularly associated with PID-5 negative affectivity, LPFS self-direction with PID-5 disinhibition, LPFS empathy with PID-5 antagonism, and LPFS intimacy with PID-5 detachment. The present study aimed to meta-analytically integrate the findings of these and other studies to investigate the extent of the associations between the elements of the LPFS (i.e., the overall LPFS score, self and interpersonal functioning, and the identity, self-direction, empathy, and intimacy elements) and the DSM-5 pathological trait domains and trait facets. In addition, as it has been suggested that the associations between Criterion A and Criterion B can be reduced by replacing the DSM-5 AMPD trait model with the personality dimensions of the FFM (25, 26), a second goal of the current investigation was to examine meta-analytically existing findings on the associations between the LPFS and the FFM traits.

## Methods

This study was pre-registered on the Open Science Framework (OSF) on March 3, 2024 (https://osf.io/49rs7). A literature search was conducted on December 2, 2024, in the databases PsycINFO, Medline, Embase, and Web of Science for studies published since 2011 (the year of the publication of the LPFS). To minimize the risk of missing relevant studies, the broad search string “personality functioning” OR “personality dysfunction” OR “personality impairment” was used. The search results from the four databases were downloaded into Zotero and processed further in this reference management software. Following the removal of duplicates, the studies’ titles, abstracts, language, and publication types were screened for possible inclusion in the meta-analysis. The full texts of the remaining studies were sought through the institutional library or by contacting the authors. The available full-texts were then assessed independently by the author and a research assistant for the following inclusion criteria, as specified in the pre-registered protocol: 1) the cross-sectional correlations of the LPFS total score, self and interpersonal functioning, and/or the identity, self-direction, empathy, and intimacy elements with the DSM-5 pathological personality trait domains or facets and/or the Five-Factor Model personality dimensions are reported; alternatively, data are provided to make it possible to calculate these correlations; 2) the DSM-5 pathological personality traits were assessed using a version of the PID-5 (15); 3) an adult sample (defined as mean age of 18 years and above) was used; 4) the study was published in a peer-reviewed journal; and 5) the language of the publication was English, German, or a Scandinavian language. Consistent with the protocol, the following exclusion criteria were applied: 1) personality functioning was not assessed in accordance with the LPFS; 2) an instrument other than a version of the PID-5 was used to assess the DSM-5 pathological personality traits; 3) adolescent samples; 4) review papers, case studies, vignette studies, and qualitative studies; and 5) unavailable publication. When reviewing the full-text studies, it became clear that the inclusion and exclusion criteria needed to be extended and refined, and the following exclusion criteria were added: 6) measurement of the LPFS with a method other than clinical assessment, interview, or self-report; 7) sample overlap with a previous study; 8) reporting adjusted correlations; and 9) using state measurement of personality functioning.

Beyond the bivariate correlations between the measures of the LPFS and the DSM-5 and FFM personality traits, study characteristics were extracted from the included publications, i.e., the publication year, country, sample size, sample type (non-clinical, clinical, or mixed), mean age, percentage of female participants, and the instruments used to assess the LPFS, DSM-5 pathological personality traits, and the FFM personality traits. A sample was classified as clinical when participants were recruited at a clinic or hospital or reported being currently in treatment for mental health problems. A research assistant checked a random selection of approximately 20% of the data for the correct extraction from the publications.

The bivariate correlations of the LPFS total score, self and interpersonal functioning, and the LPFS elements with the DSM-5 personality trait domains and facets and with the FFM personality traits were meta-analytically pooled when data from at least five independent samples were available. In studies reporting more than one correlation coefficient between scales measuring the same LPFS construct and a personality trait from the same sample, these correlations were averaged using Fisher’s z-transformation. A univariate random-effects approach, including Fisher’s z-transformation of the correlations, was used. Between-study variance was calculated using restricted maximum likelihood estimation. The 95% confidence intervals (CIs) around the pooled effects were calculated using the Hartung–Knapp method. Weighted average correlations of .30 or above were interpreted as medium and .50 or above as large (27). The *I* ^2^ statistic (28) was used to assess study heterogeneity, with values of 25%, 50%, and 75% indicating low, moderate, and high heterogeneity, respectively (28). Study characteristics (i.e., clinical vs. non-clinical sample, mean age, proportion of female participants, and self-reported vs. clinician-rated personality functioning) were individually examined as potential moderators of the pooled correlations in a series of meta-regressions. The analyses were conducted only when at least 10 studies were available (cf. 29). Given the large number of tests, the statistical significance level was set at *p* <.01.

The statistical analyses were conducted in R (version 4.5.1; 30) and the packages misty (version 0.7.2; 31), psych (version 2.5.6; 32), and meta (version 8.1-0; 33) to calculate correlations from provided data sets, perform Fisher’s z-transformations, and conduct the meta-analyses, respectively.

## Results

**Figure 1:**
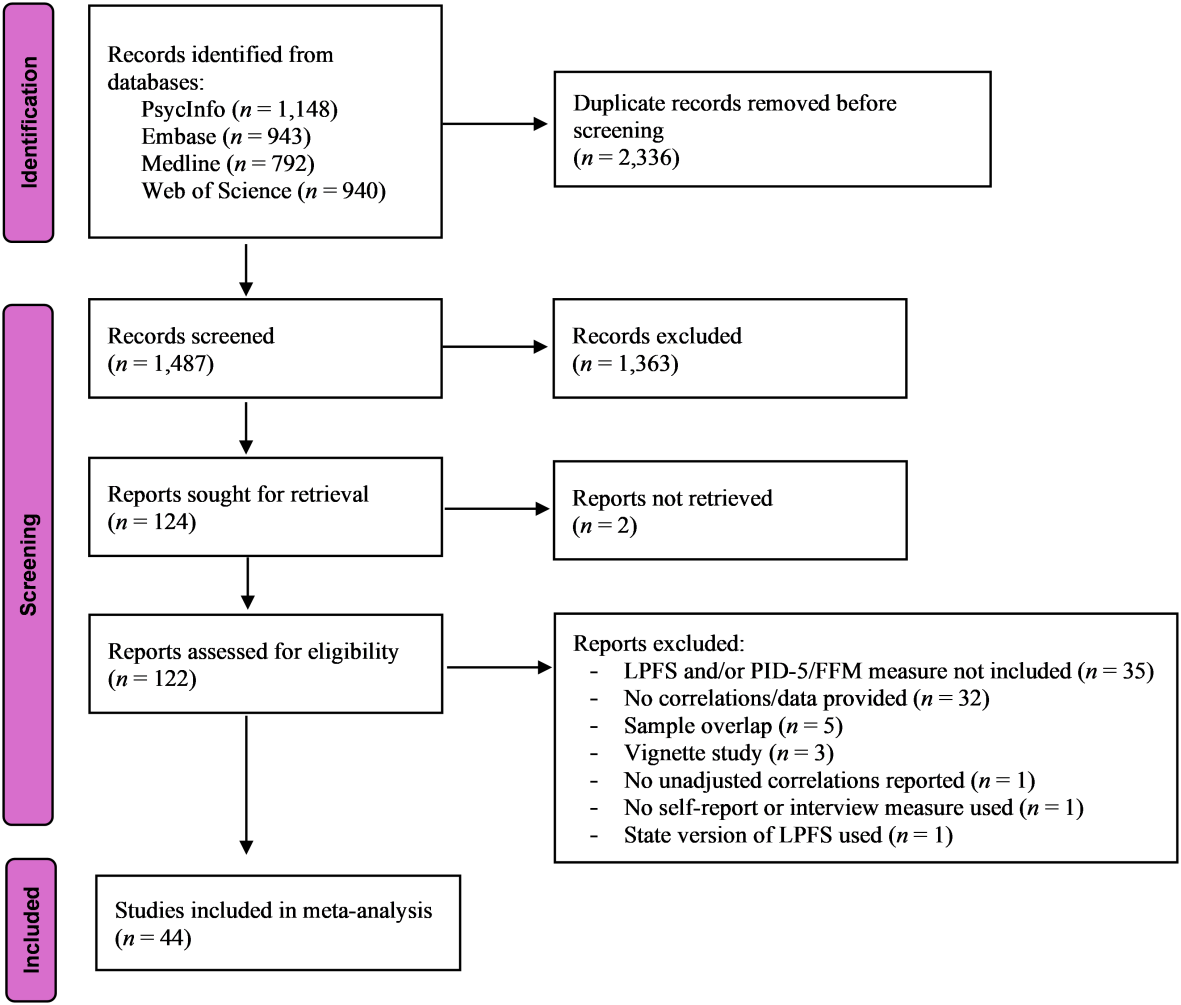
PRISMA flowchart of the study selection process. Since all studies were identified from databases, the right part of the PRISMA (34) flow diagram (identification of studies via other methods) is not displayed.

A Preferred Reporting Items for Systematic reviews and Meta-Analyses (PRISMA) flowchart depicting the search process for eligible studies is shown in **Figure 1**. The four databases yielded 1,487 unique search results. The screening of the entries resulted in 124 publications for assessment in full-text, two of which could not be retrieved. Forty-four publications comprising 48 independent samples met the eligibility criteria. The supplementary data files for two samples contained personally identifiable information (location and IP address) and were not analyzed in the present study to comply with European personal data protection regulations prohibiting the processing of personal data without consent. For one sample, the information provided in the published article was used (24), while the other sample was excluded from the investigation [the undergraduate sample of the Sorem, Priebe, and Anderson (35) study]. Thus, data from 44 studies and 47 samples were included in the analyses. **Table 1** provides an overview of the study and sample characteristics. Most studies were conducted in Western countries. The majority of samples were non-clinical (*n* = 27), 13 samples were clinical, and seven samples were categorized as mixed clinical and non-clinical. The sample sizes ranged from *n* = 88 to *n* = 3,019, the mean age of the samples ranged from 18.79 to 67.46 years, and the proportion of female participants ranged from 24.8% to 81.7%. A variety of measures were used to assess the LPFS, most frequently the Level of Personality Functioning Scale-Brief Form (LPFS-BF; 75, 76) and the Level of Personality Functioning Scale-Self-Report (LPFS-SR; 77). The original PID-5 was the most frequently administered version of the instrument (**Table 1**).

**Table 1:** Characteristics of the studies included in the meta-analysis.

Study	Country	Sample size	Sample type	Mean age (years)	% Female	LPFS instrument	Version of PID-5	FFM instrument
Amini et al. (36)	Iran	290	Mixed	40.0	24.8	LPFS-BF 2.0	PID-5-BF	
Baaijens et al. (37)	Netherlands	242	Clinical	18.79	73	LPFS-BF 2.0	PID-5-SF	
Bach and Hutsebaut (38)	Denmark	228	Clinical	31.5	42	LPFS-BF 2.0	PID-5-SF	
Bliton et al. (39)	USA	608	Non-clinical	19.38	51.2	LPFS-SR, LPFS-SRA, LPFS-BF		HEXACO-60
Bottesi et al. (40)	Italy	1,183	Non-clinical	31.3	46.2	DLOPFQ-SF	PID-5	
Chamandoost et al. (41)	Iran	496	Non-clinical	34.0	68.5	LPFS-SR	PID-5-BF	NEO-FFI
Cruitt et al. (42)	USA	162	Non-clinical	–	56.2	LPFS ratings based on LSI		NEO-PI-R
Emery et al. (43)	USA	152	Clinical	43.2	63.2	LPFS clinician-rated	PID-5	BFI
Few et al. (21)	USA	109	Clinical	35.9	71	LPFS clinician-rated	PID-5	
Gamache et al. (44)	Canada	386	Mixed	31.6	70.7	SIFS	PID-5-SF	
Haehner et al. (45)	Germany	1,151	Non-clinical	29.33	75	LPFS-BF 2.0		BFI-2-XS
Halberstadt et al. (46)	USA	500	Non-clinical	52.28	50	LPFS-SR-BF	PID-5-SF	
Hessels et al. (47)	Netherlands	246	Clinical	19.2	81.7	LPFS-BF 2.0	PID-5-BF	
Hopwood et al. (24)	USA	1,976	Non-clinical	34.98–35.47	45 and 47	LPFS-SR	PID-5	BFI-2
Huprich et al. (48)	USA	140	Clinical	47.79	73.6	DLOPFQ	PID-5-BF	
Katar et al. (49)	Turkey	220	Clinical	37.23	78.2	LPFS clinician-rated	PID-5	
Labonte and Kealy (50)	Canada	393	Non-clinical	34.26	69.5	LPFS-BF 2.0		Mini-IPIP
Lakuta et al. (51, 52)	Poland	242	Non-clinical	30.63	52.9	LPFS-SR, LPFS-BF 2.0	PID-5	BFI-2
Leclerc et al. (53)	Canada	285	Clinical	33.72	62.1	SIFS	PID-5-SF	
		995	Non-clinical	46.16	76.8	SIFS	PID-5-SF	
Li et al. (54)	China	3,019	Non-clinical	19.45	57.8	LPFS-BF 2.0	PID-5	
Lorentzen et al. (55)	Norway	295	Non-clinical	30.0	75.9	LPFS-BF 2.0	PID5BF+M	
Macina et al. (56)	Germany/Switzerland	886	Mixed	37.2	48	SIFS	PID5BF+M	
McCabe and Widiger (57)	USA	300	Non-clinical	36.51	54	LPFS-SR	PID-5	
McCabe et al. (58)	USA	402	Mixed	33.41	55	LPFS-BF, AS-LPFS, DLOPFQ, LPFS-SR		IPIP-NEO-120, BFI
Natoli et al. (59)	USA	371	Non-clinical	21.33	77.26	LPFS-BF 2.0	PID5BF+	BFI-2-S
Nysaeter et al. (22)	Norway	317	Mixed	32.2	65.2	SCID-5-AMPD-I	PID-5	
Ohse et al. (60)	Germany	121	Clinical	32.2	71.1	SCID-5-AMPD-I, LPFS-SR	PID-5-SF	
Olivera et al. (61)	Brazil	415	Non-clinical	29.31	74.7	LPFS-BF 2.0	PID-5-SF	
		1,011	Non-clinical	31.66	72.1	LPFS-BF 2.0	PID5BF+M	
Oltmanns and Widiger (62)	USA	269	Mixed	31.8	68	LPFS-SR		Composite of IPIP-NEO-120, FFF, and FFMRF
Pires et al. (63)	Portugal	280	Non-clinical	48.01	53.2	LPFS-SR	PID5BF+M	
		131	Clinical	42.66	45	LPFS-SR	PIF5BF+M	
Riegel et al. (64)	Czechia	421	Clinical	39.3	30.6	LPFS-SR	PIF5BF+M	
		567	Non-clinical	32.9	65.2	LPFS-SR	PIF5BF+M	
Roche and Jaweed (65)	USA	204	Non-clinical	18.9	69	LPFS-SRA, LPFS-SR, LPFS-BF 2.0, SIFS, DLOPFQ	PID-5-BF	
Rossi and Diaz-Batanero (66)	Spain	1,074	Mixed	43.45	50.3	LPFS-BF 2.0	PID-5-SF	
Savard et al. (67)	Canada	350	Clinical	35.44	62.3	SIFS	PID-5-SF	
Sleep et al. (23)	USA	308	Non-clinical	35.77	67	LPFS-SR	PID-5-SF	
Sleep et al. (68)	USA	365	Non-clinical	19.07	58	LPFS-SR	PID-5-SF	IPIP-120
Somma et al. (69)	Italy	88	Clinical	36.47	54.5	SCID-5-AMPD-I, LPFS-SR, LPFS-BF	PID-5	
Sorem et al. (35)	USA	278	Non-clinical	36.7	59.3	LPFS-SR	PID-5	
Stone and Segal (70)	USA	202	Non-clinical	67.46	39	LPFS-SR	PID-5	
Stone et al. (71)	USA	130	Non-clinical	64.61	65	LPFS-BF 2.0		BFI-2
Stover et al. (72)	Argentine	342	Non-clinical	39.9	50	PFS	PID-5-BF	
Strand et al. (73)	Norway	1,278	Non-clinical	29.5	51.6	LPFS-BF 2.0	PIF5BF+M	BFI-10
Valls et al. (74)	Sweden	253	Non-clinical	–	61	LPFS-BF 2.0	PID-5-BF	

AS-LPFS, Anderson and Sellbom Level of Personality Functioning Scale; BFI, Big Five Inventory; BFI-2, Big Five Inventory-2; BFI-2-S, Big Five Inventory-2 Short Form; BFI-2-XS, Big Five Inventory-2 Extra-Short Form; DLOPFQ, DSM-5 Levels of Functioning Questionnaire; DLOPFQ-SF, DSM-5 Levels of Functioning Questionnaire-Short Form; FFF, Five-Factor Form; FFM, Five-Factor Model of personality; FFMRF, Five-Factor Model Rating Form; IPIP-NEO-120, International Personality Item Pool-NEO-120; LPFS, Level of Personality Functioning Scale; LPFS-BF, Level of Personality Functioning Scale-Brief Form; LPFS-SR, Level of Personality Functioning Scale-Self-Report; LPFS-SR-BF, Level of Personality Functioning Scale-Self-Report Brief Form; LPFS-SRA, Level of Personality Functioning Scale Self-Report of Criterion A; LSI, Life Story Interview; Mini-IPIP, Mini-International Personality Item Pool—Five-Factor Model Scale; NEO-FFI, NEO Five-Factor Inventory; NEO-PI-R, NEO Personality Inventory-Revised; PID-5, Personality Inventory for DSM-5; PID-5-BF, Personality Inventory for DSM-5-Brief Form; PID5BF+M, Modified Personality Inventory for DSM-5 and ICD-11–Brief Form Plus; PID-5-SF, Personality Inventory for DSM-5-Short Form; PFS, Personality Functioning Scale; SCID-5-AMPD-I, Structured Clinical Interview for the DSM-5 Alternative Model for Personality Disorders Module I; SIFS, Self and Interpersonal Functioning Scale.

The results of the meta-analyses of the correlations of scales assessing the LPFS with PID-5 scales are shown in **Table 2**. Study heterogeneity was high in almost all analyses. The weighted average correlation coefficients between the LPFS total score and the PID-5 trait domains ranged from .44 (antagonism) to .64 (detachment), with all correlations indicating large effect sizes (≥.50) except for antagonism. The mean correlations between the LPFS total score and the PID-5 trait facets were mostly in the range of medium to large effect sizes, varying from .25 (attention-seeking) to .68 (depressivity). Thirteen of the 25 average correlation coefficients were larger than .50. In addition to depressivity, the anhedonia, emotional lability, perseveration, and suspiciousness scales had mean correlations above .60 with the LPFS total score.

**Table 2:** Meta-analytically pooled correlations between LPFS measures and PID-5 scales.

PID-5 trait domains and facets	LPFS total (95% CI)	Self (95% CI)	Interpersonal (95% CI)	Identity (95% CI)	Self-direction (95% CI)	Empathy (95% CI)	Intimacy (95% CI)
Negative affectivity	.63 (.59, .67)	.60 (.53, .67)	.46 (.40, .52)	.66 (.62, .70)	.53 (.48, .57)	.41 (.37, .44)	.45 (.39, .49)
*k*, *I* ^2^	29, 88.2%	13, 90.4%	13, 80.7%	20, 86.0%	20, 82.7%	20, 64.0%	20, 83.4%
Detachment	.64 (.60, .68)	.54 (.47, .61)	.56 (.50, .61)	.55 (.50, .59)	.48 (.44, .52)	.46 (.41, .50)	.61 (.56, .65)
*k*, *I* ^2^	29, 90.6%	13, 90.0%	13, 80.9%	21, 88.3%	21, 80.5%	21, 80.1%	21, 91.6%
Antagonism	.44 (.37, .50)	.35 (.25, .44)	.45 (.37, .52)	.30 (.26, .34)	.33 (.29, .37)	.43 (.39, .46)	.34 (.29, .38)
*k*, *I* ^2^	29, 91.8%	13, 91.4%	13, 86.3%	21, 74.6%	21, 81.7%	21, 72.8%	21, 81.6%
Disinhibition	.55 (.48, .60)	.49 (.40, .56)	.45 (.38, .52)	.49 (.44, .54)	.55 (.50, .60)	.42 (.37, 47)	.37 (.31, .42)
*k*, *I* ^2^	29, 92.9%	13, 91.2%	13, 84.2%	20, 85.1%	20, 83.5%	20, 83.0%	20, 83.0%
Psychoticism	.57 (.52, .62)	.55 (.49, .60)	.52 (.47, .57)	.49 (.45, .53)	.43 (.39, .47)	.46 (.42, .50)	.43 (.39, .47)
*k*, *I* ^2^	29, 91.8%	13, 86.7%	13, 80.3%	21, 83.3%	21, 79.6%	21, 81.6%	21, 73.7%
Anhedonia	.63 (.55, .71)			.58 (.48, .66)	.57 (.47, .65)	.44 (.35, .52)	.51 (.42, .59)
*k*, *I* ^2^	8, 89.3%			7, 88.2%	7, 86.9%	7, 74.9%	7, 80.7%
Anxiousness	.57 (.51, .63)			.59 (.53, .65)	.45 (.37, .53)	.35 (.27, .43)	.43 (.32, .53)
*k*, *I* ^2^	9, 90.7%			8, 83.0%	8, 91.1%	8, 91.2%	8, 88.2%
Attention seeking	.25 (.14, .36)			.23 (.07, .38)	.26 (.14, .38)	.24 (.10, .37)	.19 (.00, .37)
*k*, *I* ^2^	6, 90.3%			5, 89.8%	5, 87.1%	5, 90.7%	5, 94.3%
Callousness	.44 (.25, .60)			.33 (.10, .53)	.38 (.12, .59)	.57 (.44, .69)	.51 (.33, .65)
*k*, *I* ^2^	6, 98.1%			5, 96.8%	5, 97.6%	5, 94.1%	5, 96.3%
Cognitive and perceptual dysregulation	.50 (.35, .63)			.42 (.24, .56)	.44 (.26, .59)	.44 (.27, .58)	.34 (.14, .52)
*k*, *I* ^2^	8, 97.1%			7, 97.4%	7, 97.7%	7, 96.8%	7, 97.5%
Deceitfulness	.42 (.33, .51)			.37 (.25, .47)	.39 (.26, .50)	.42 (.30, .53)	.38 (.24, .50)
*k*, *I* ^2^	8, 93.3%			7, 90.7%	7, 90.7%	7, 91.2%	7, 92.9%
Depressivity	.68 (.62, .73)			.66 (.56, .74)	.62 (.53, .69)	.47 (.38, .56)	.54 (.48, .60)
*k*, *I* ^2^	7, 87.0%			6, 92.0%	6, 88.5%	6, 83.7%	6, 73.7%
Distractibility	.57 (.49, .64)			.53 (.44, .61)	.52 (.42, .61)	.39 (.29, .48)	.38 (.25, .48)
*k*, *I* ^2^	8, 87.9%			7, 91.4%	7, 92.9%	7, 89.8%	7, 91.9%
Eccentricity	.55 (.45.64)			.47 (.34, .58)	.41 (.30, .51)	.45 (.36, .53)	.44 (.29, .56)
*k*, *I* ^2^	8, 91.7%			7, 94.4%	7, 91.3%	7, 84.6%	7, 91.9%
Emotional lability	.61 (.53, .68)			.61 (.54, .68)	.50 (.41, .59)	.42 (.31, .51)	.46 (.33, .56)
*k*, *I* ^2^	9, 92.9%			8, 89.6%	8, 89.0%	8, 90.6%	8, 91.9%
Grandiosity	.31 (.23, .38)			.20 (.10, .30)	.23 (.15, .31)	.40 (.31, .48)	.32 (.22, .40)
*k*, *I* ^2^	8, 78.1%			7, 80.1%	7, 75.2%	7, 71.4%	7, 82.9%
Hostility	.59 (.50, .67)			.54 (.42, .63)	.47 (.35, .57)	.49 (.39, .59)	.48 (.36, .59)
*k*, *I* ^2^	7, 94.2%			6, 93.1%	6, 92.6%	6, 90.5%	6, 92.7%
Impulsivity	.52 (.42, .61)			.45 (.32, .56)	.52 (.42, .61)	.43 (.31, .53)	.37 (.26, .48)
*k*, *I* ^2^	9, 95.1%			8, 95.2%	8, 91.2%	8, 92.8%	8, 93.2%
Intimacy avoidance	.38 (.26, .49)			.29 (.18, .39)	.27 (.11, .43)	.30 (.13, .45)	.39 (.27, .49)
*k*, *I* ^2^	8, 89.2%			7, 85.7%	7, 92.4%	7, 93.2%	7, 89.9%
Irresponsibility	.52 (.42, .61)			.43 (.26, .57)	.52 (.39, .63)	.45 (.33, .56)	.42 (.28, .54)
*k*, *I* ^2^	8, 95.2%			7, 95.1%	7, 94.9%	7, 94.1%	7, 95.0%
Manipulativeness	.26 (.21, .31)			.20 (.10, .30)	.27 (.23, .30)	.27 (.21, .33)	.24 (.15, .32)
*k*, *I* ^2^	8, 64.6%			7, 80.2%	7, 0.0%	7, 57.0%	7, 80.3%
Perseveration	.66 (.61, .70)			.60 (.47, .71)	.58 (.47, .68)	.50 (.38, .60)	.50 (.34, .63)
*k*, *I* ^2^	6, 69.1%			5, 92.1%	5, 89.0%	5, 87.5%	5, 93.0%
Restricted affectivity	.28 (.18, .37)			.16 (.01, .29)	.22 (.08, .36)	.33 (.20, .44)	.30 (.19, .40)
*k*, *I* ^2^	6, 86.9%			5, 90.8%	5, 90.8%	5, 84.4%	5, 84.4%
Rigid perfectionism (lack of)	.38 (.29, .46)			.35 (.24, .44)	.23 (.12, .34)	.27 (.14, .39)	.31 (.19, .43)
*k*, *I* ^2^	8, 75.7%			7, 76.6%	7, 80.9%	7, 85.3%	7, 87.5%
Risk taking	.35 (.19, .49)			.26 (.06, .44)	.34 (.21, .46)	.34 (.20, .47)	.30 (.14, .44)
*k*, *I* ^2^	7, 98.1%			6, 98.2%	6, 96.7%	6, 96.6%	6, 97.1%
Separation insecurity	.47 (.40, .53)			.49 (.42, .56)	.42 (.34, .50)	.36 (.26, .45)	.37 (.28, .45)
*k*, *I* ^2^	9, 91.1%			8, 86.3%	8, 88.2%	8, 90.1%	8, 88.8%
Submissiveness	.43 (.39, .48)			.47 (.45, .48)	.40 (.37, .43)	.26 (.17, .35)	.28 (.24, .32)
*k*, *I* ^2^	6, 45.9%			5, 0.0%	5, 0.0%	5, 69.0%	5, 0.0%
Suspiciousness	.64 (.55, .71)			.57 (.46, .67)	.47 (.35, .58)	.57 (.44, .68)	.62 (.50, .72)
*k*, *I* ^2^	6, 80.6%			5, 82.4%	5, 80.5%	5, 86.5%	5, 87.5%
Unusual beliefs and experiences	.46 (.35, .55)			.38 (.24, .50)	.37 (.23, .48)	.37 (.24, .49)	.36 (.20, .50)
*k*, *I* ^2^	8, 92.7%			7, 93.9%	7, 93.9%	7, 94.4%	7, 95.6%
Withdrawal	.56 (.51, .60)			.48 (.44, .52)	.43 (.38, .47)	.45 (.39, .51)	.59 (.49, .67)
*k*, *I* ^2^	8, 47.5%			7, 46.8%	7, 29.6%	7, 56.9%	7, 81.4%

LPFS, Level of Personality Functioning Scale; PID-5, Personality Inventory for DSM-5.

LPFS self-functioning scales showed a large effect size mean correlations with the PID-5 trait domains negative affectivity, psychoticism, and detachment. For LPFS interpersonal functioning scales, the average correlations with detachment and psychoticism reached the threshold of .50. All other correlation coefficients were in the medium range. Since fewer than five studies reported the correlations between LPFS self and interpersonal functioning scales and the PID-5 trait facets, meta-analyses on these associations were not performed. However, as these studies provided correlations between other LPFS and PID-5 scales, they were retained in the meta-analysis.

The measures of LPFS identity were most strongly correlated with the PID-5 trait domains of negative affectivity and detachment, with large effect sizes. Concerning the PID-5 trait facets, large-sized average correlations were found with (in descending order of their size) depressivity, emotional lability, perseveration, anxiousness, anhedonia, suspiciousness, hostility, and distractibility. LPFS self-direction measures showed the highest pooled correlations with the PID-5 trait domains disinhibition and negative affectivity and the trait facets depressivity, perseveration, anhedonia, distractibility, impulsivity, irresponsibility, and emotional lability, with large effect sizes. The average correlations between LPFS empathy scales and the PID-5 trait domains showed little variation and ranged from .41 (negative affectivity) to .46 (psychoticism). On the trait facet level, LPFS empathy scales were most strongly correlated with callousness, suspiciousness, and perseveration (all three correlations were .50 or above). For the measures of LPFS intimacy, pooled large-sized correlations were found with the PID-5 trait domain detachment and the trait facets of suspiciousness, withdrawal, depressivity, callousness, anhedonia, and perseveration.

Meta-regression analyses using study characteristics as moderators were conducted for the correlations between the LPFS scales and the PID-5 trait domains. Only for the association between the LPFS total score and disinhibition was a moderating effect of the mean age of the participants in the samples found (*b* = 0.01, *p* = .006).

**Table 3** shows the weighted average correlations between the measures of the LPFS and the FFM. All pooled correlations with neuroticism were positive, while the associations with extraversion, openness, agreeableness, and conscientiousness were negative. With only a few exceptions, study heterogeneity was high in the analyses. The LPFS total score was most strongly correlated with neuroticism, with a coefficient close to the threshold for a large effect size (.49). The correlations with extraversion, agreeableness, and conscientiousness were medium-sized, while the association with openness was small, and the 95% CI for the correlation coefficient included zero. The measures of LPFS self-functioning showed a large correlation with neuroticism, medium correlations with extraversion, agreeableness, and conscientiousness, and a small correlation with openness. Scales assessing LPFS interpersonal functioning had their highest pooled correlations with agreeableness and neuroticism in the medium-to-large range. The average correlations with the remaining FFM dimensions were small to medium. The measures of LPFS identity had a large-sized correlation with neuroticism, medium-sized correlations with extraversion, agreeableness, and conscientiousness, and were unrelated to openness (the 95% CI for the correlation coefficient included zero). Scales assessing the LPFS self-direction element showed the strongest pooled correlations with neuroticism and conscientiousness, with medium-to-large effect sizes. The LPFS empathy element had its largest combined correlation with agreeableness (−.42), and the intimacy element with neuroticism (.41) and agreeableness (−.39).

**Table 3:** Meta-analytically pooled correlations between the LPFS and the FFM personality traits.

FFM traits	LPFS total (95% CI)	Self (95% CI)	Interpersonal (95% CI)	Identity (95% CI)	Self-direction (95% CI)	Empathy (95% CI)	Intimacy (95% CI)
Neuroticism	.49 (.35, .60)	.64 (.59, .67)	.44 (.37, .50)	.56 (.41, .68)	.45 (.32, .55)	.28 (.17, .38)	.41 (.33, .48)
*k*, *I* ^2^	10, 95.2%	6, 63.4%	6, 60.1%	8, 93.3%	8, 86.2%	8, 79.5%	8, 71.6%
Extraversion	−.25 (−.39, −.09)	−.29 (−.40, −.18)	−.21 (−.32, −.10)	−.24 (−.33, −.15)	−.24 (−.33, −.14)	−.11 (−.21, −.02)	−.26 (−.31, −.21)
*k*, *I* ^2^	10, 97.8%	6, 88.8%	6, 83.0%	8, 84.2%	8, 87.0%	8, 82.8%	8, 41.7%
Openness	−.07 (−.18, .03)	−.17 (−.28, −.06)	−.26 (−.40, −.12)	−.06 (−.17, .04)	−.15 (−.27, −.02)	−.16 (−.27, −.04)	−.13 (−.23, −.04)
*k*, *I* ^2^	10, 96.2%	6, 88.2%	6, 91.1%	8, 90.9%	8, 93.8%	8, 91.6%	8, 89.6%
Agreeableness	−.29 (−.44, −.13)	−.35 (−.49, −.20)	−.46 (−.55, −.35)	−.31 (−.40, −.21)	−.35 (−.44, −.25)	−.42 (−.49, −.34)	−.39 (−.48, −.29)
*k*, *I* ^2^	10, 98.5%	6, 93.0%	6, 92.9%	8, 88.5%	8, 84.8%	8, 85.7%	8, 90.8%
Conscientiousness	−.30 (−.47, −.12)	−.41 (−.57, −.21)	−.32 (−.42, −.22)	−.36 (−.44, −.26)	−.44 (−.55, −.32)	−.28 (−.35, −.19)	−.28 (−.36, −.19)
*k*, *I* ^2^	10, 98.7%	6, 97.4%	6, 88.3%	8, 86.8%	8, 91.5%	8, 72.4%	8, 82.2%

LPFS, Level of Personality Functioning Scale; FFM, Five-Factor Model.

## Discussion

The distinction between the severity and style of personality pathology in Criterion A and Criterion B in the diagnosis of PDs is arguably the most innovative feature of the DSM-5 AMPD. The models and measures of self and interpersonal functioning (the LPFS) and pathological personality traits (the PID-5) have been developed to operationalize these new criteria. Since the publication of the DSM-5 AMPD, the relationship between the LPFS and the trait model has become a topic of research and debate. The aim of the present study was to use a meta-analytical approach to examine the bivariate associations of LPFS measures with the PID-5 trait domains and trait facets, as well as the FFM personality dimensions. Data from 47 independent samples were identified through a systematic literature search and analyzed in this investigation. Overall, the results showed medium-to-high correlations between the LPFS and the PID-5 pathological trait domains and trait facets. More specifically, the weighted average correlations between the LPFS total score and the PID-5 trait domains ranged from .44 (antagonism) to .64 (detachment). More than half of the PID-5 trait facet scales exhibited high correlations (.50 or above) with the overall LPFS score. Medium- to large-sized correlations were also found for self and interpersonal functioning and the LPFS elements identity, self-direction, empathy, and intimacy with pathological personality traits. Self-functioning was the most strongly related to negative affectivity, and interpersonal functioning to detachment. Identity was highly correlated with negative affectivity and detachment, self-direction with disinhibition and negative affectivity, empathy with callousness and suspiciousness, and intimacy with detachment. The perseveration, depressivity, anhedonia, and depression trait facets showed strong relationships with several LPFS elements. No moderating effects of study characteristics on the associations between the LPFS and the PID-5 trait domains were observed, except that mean sample age moderated the correlation between the LPFS total score and disinhibition, indicating that correlations increased with age. The size of the pooled correlations was generally lower when the associations of the LPFS with the FFM dimensions were examined, but often still in the medium-to-large range, especially for neuroticism and agreeableness.

The results of this study showed substantial relationships between the LPFS and the pathological personality traits of the DSM-5 AMPD. Most correlations between scales assessing the LPFS and the PID-5 trait domains and trait facets were at least medium-sized, and many correlations had large effect sizes. Unfortunately, the study provides no explanation for the considerable relationships. It appears, however, likely that several factors, alone or in combination, may play a role. First, the pattern of sizeable associations across a wide range of constructs suggests an effect of non-specific factors. For example, general personality dysfunction is common to the LPFS and pathological personality traits (78) and may partly account for their associations. This interpretation is supported by the findings in the present study that most PID-5 trait domains and trait facets were highly correlated with the LPFS total score, as well as by the analyses on the relationships between the LPFS and the FFM dimensions, which showed lower correlations compared to the PID-5 traits. Further, shared method variance may have inflated the associations, as most studies included in the meta-analysis assessed the LPFS with a self-report instrument. Several large-sized correlations appear to reflect the similarity of the content of the LPFS and the PID-5 traits, e.g., between LPFS identity and PID-5 depressivity, as both encompass self-esteem. Another explanation for the observed associations can be that there are causal relationships between personality functioning and pathological personality traits. For example, it has been suggested that the elements of personality functioning underlie and drive trait manifestations (79) or that they can be thought of as capacities and that pathological traits represent the individual’s tendency to not behave in accordance with these capacities (80, 81). For example, the trait callousness can be understood as resulting from an impairment in the capacity for empathy (81). Vice versa, some authors have argued that impaired personality functioning is the result of maladaptive personality traits (e.g., 82). From this perspective, the observed association between LPFS intimacy and PID-5 detachment can, for example, be explained by trait detachment—particularly the withdrawal and suspiciousness facets—causing impairment in the LPFS intimacy element. Trait perseveration, which in the present study was strongly related to all LPFS elements, could be understood as a general underlying factor affecting all aspects of personality functioning. Finally, the distinctiveness of personality functioning and personality traits may have been blurred by the operationalization of these constructs by incorporating items assessing personality traits in LPFS instruments and including items covering personality functioning (e.g., identity) in the measures of personality traits (83). Given that the associations between personality functioning and pathological personality traits are well-documented, future research should focus on understanding and clarifying the nature of these relationships.

As a consequence of the observed associations between the LPFS and pathological personality traits, some authors have argued that Criterion A of the DSM-5 AMPD should be abandoned and that the assessment of personality traits is sufficient to determine the severity of personality pathology (e.g., 84, 85). However, the presence of pathological personality traits is not limited to PDs and can be found in other forms of psychopathology to the same or even higher degree than in PDs (86). Moreover, despite substantial correlations, it is not clear whether LPFS and PID-5 constructs can and should be considered identical and interchangeable. Drawing on McAdams’ theory of personality (87) and personality development (88), it has been proposed that identity in terms of the individuals’ subjective meaning-making of itself and its life history is conceptually distinct from personality traits and necessary to incorporate in PD assessment to fully understand personality functioning (4, 89, 90). Moreover, it has been suggested that the LPFS and its elements are more aligned with clinicians’ thinking about PDs and more easily integrated into existing psychological treatments than pathological personality traits (e.g., 91). Finally, to retain the distinction between severity and style of personality pathology but to reduce the empirical associations, the definition and operationalization of personality functioning and personality traits can be revised and refined. For example, the LPFS can be modified to focus on the capacity to mentalize oneself and others (cf., 4). On the other hand, the maladaptive trait model of the DSM-5 AMPD can be replaced by the normal personality dimensions of the FFM (25, 26). In addition to weaker associations with the LPFS, normal-range personality traits have shown higher stability than maladaptive personality traits and cover more variants of personality due to their bipolarity than the unidimensional PID-5 personality traits (3, 25). However, replacing the PID-5 traits with FFM dimensions will not eliminate the associations between Criterion A and Criterion B. The current study’s findings suggest smaller but still moderate correlations between the LPFS and the FFM traits. Given the associations of normal-range personality traits, especially neuroticism, with psychopathology (92, 93), it has to be expected, that the severity and style of personality pathology are related to some degree.

The current investigation has limitations that must be taken into consideration when interpreting its results. First, the study examined the bivariate associations between the LPFS and the PID-5 personality traits. However, to estimate the overall associations between the LPFS and pathological personality traits, multivariate analyses are needed. For example, predicting the LPFS total score from all PID-5 trait domains provides an estimate of the proportion of variance in the LPFS that the trait domains explain combined and information about the unique contributions of the individual traits by controlling for the intercorrelations between the PID-5 traits (15). Next, the LPFS was assessed using a variety of instruments, which may have increased the heterogeneity between studies. More importantly, factorial evaluations of some multidimensional LPFS measures have found problems with the proposed internal structures, i.e., the distinction between self and interpersonal functioning and between the identity, self-direction, empathy, and intimacy elements (56, 68, 94). Further, a lack of discriminant validity of scales designed to assess the LPFS has been found (23, 58). The precision of the effect size estimates obtained in a meta-analysis is naturally affected by the number of studies included in the analysis. While there was a moderate number of data publicly available on the associations between the LPFS and the PID-5 trait domains, few studies have investigated the PID-5 trait facets and the FFM traits, resulting in large confidence intervals in the results of the meta-analyses. In addition, the limited number of studies reduced the statistical power to detect moderators of the observed associations. Finally, most studies included in this meta-analysis used non-clinical samples composed primarily of female young adults and were conducted in Western countries. However, the usefulness and applicability of the DSM-5 AMPD across different sociocultural contexts are unclear (95). Thus, the generalizability of the study’s findings to clinical samples and to more diverse populations in terms of age, gender, and culture is uncertain.

In conclusion, the results of the present study suggest medium-to-large-sized associations between the LPFS and the PID-5 trait domains and trait facets in the DSM-5 AMPD. Sharing personality dysfunction, similar constructs, causal connections, and shared method variance may each contribute to these associations to unknown degrees. The correlations of the LPFS with the FFM dimensions were lower than those with the PID-5 traits. The magnitude of the associations between Criterion A and Criterion B suggests that the two criteria should be modified for a more efficient assessment of the DSM-5 AMPD.

## Data Availability

Publicly available datasets were analyzed in this study. This data can be found here: https://osf.io/xvbwg/, https://osf.io/e35ug/, https://hdl.handle.net/10272/21157, https://osf.io/bhq94/, https://osf.io/49sdc/.
